# Interspecific coral competition does not affect the symbiosis of gall crabs (Decapoda: Cryptochiridae) and their scleractinian hosts

**DOI:** 10.1002/ece3.10051

**Published:** 2023-05-11

**Authors:** Susanne Bähr, Sancia E. T. van der Meij, Tullia I. Terraneo, Tao Xu, Francesca Benzoni

**Affiliations:** ^1^ Marine Science Program Biological and Environmental Science and Engineering Division King Abdullah University of Science and Technology (KAUST) Thuwal Saudi Arabia; ^2^ KAUST Red Sea Research Center King Abdullah University of Science and Technology Thuwal Saudi Arabia; ^3^ Groningen Institute for Evolutionary Life Science (GELIFES) University of Groningen Groningen The Netherlands; ^4^ Marine Biodiversity Group Naturalis Biodiversity Center Leiden The Netherlands

**Keywords:** associated fauna, coral reefs, host‐specificity, *Pavona*, *Porites*

## Abstract

Coral reefs accommodate a myriad of species, many of which live in association with a host organism. Decapod crustaceans make up a large part of this associated fauna on coral reefs. Among these, cryptochirid crabs are obligately associated with scleractinian corals, in which they create dwellings where they permanently reside. These gall crabs show various levels of host specificity, with the majority of cryptochirids inhabiting a specific coral genus or species. Here, we report the first records of gall crabs living in association with two different *Porites* species in the Red Sea. Crescent‐shaped dwellings were observed in *Porites rus* and a *Porites* sp. in situ, and colonies with crabs were collected for further study in the laboratory. Using a combination of morphology and DNA barcoding, the crabs were identified as belonging to *Opecarcinus*, a genus only known to inhabit Agariciidae corals. The coral skeleton was bleached and studied under a stereo microscope, which revealed that the *Porites* corals overgrew adjoining agariciid *Pavona* colonies. We hypothesize that the gall crab originally settled on *Pavona*, its primary host of choice. Due to coral interspecific competition the *Porites* colony overgrew the adjacent *Pavona* colonies, resulting in a secondary and never before reported association of *Opecarcinus* with *Porites*. These findings suggest that cryptochirid crabs can adapt to the new microenvironment provided by a different coral host and survive competition for space on coral reefs.

## Graphical abstract

We report the first observation of coral‐dwelling gall crabs inhabiting corals of the family Poritidae in the central Red Sea. Based on examination of the coral skeletons containing the crab dwellings, we hypothesize that the crabs initially settled on their preferred host (Agariciidae), which was subsequently outcompeted and overgrown by the *Porites* colonies. These findings give interesting insights into the adaptive potential of gall crabs to changes in their microenvironment and their ability to survive competition for space on coral reefs.

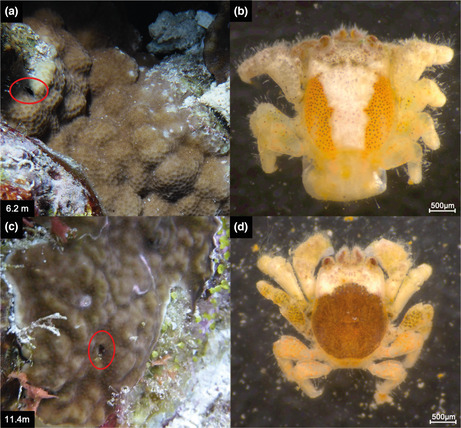

## 1 INTRODUCTION

Coral reefs are the most species‐rich marine ecosystem. Due to their high habitat heterogeneity and the abundance of sessile host organisms inhabiting coral reefs, these ecosystems house the highest diversity of symbiotic relationships in the marine environment (Fisher et al., 2015; Hoegh‐Guldberg, 1999). As major hermatypic reef builders, scleractinian corals are hosts to an extraordinary number of invertebrate species that rely on the corals for food, habitat, or refuge from predation (Castro, 1988; Paulay, 1997; Stella et al., 2011). In return, some symbionts may protect their hosts against corallivorous predators, increase coral health by clearing sediment from their hosts, or reduce their susceptibility to diseases (Montano et al., 2017; Pratchett, 2001; Rouzé et al., 2014; Stewart et al., 2006; Stier et al., 2010). Some of the most common symbionts of corals are brachyuran crabs, which often live in obligate commensal relationships with scleractinian corals (Castro, 2015). Despite exhibiting a secluded lifestyle, coral‐dwelling gall crabs (Cryptochiridae Paulson, 1875) are a noteworthy, widely distributed and highly abundant group of reef‐associated crustaceans (van Tienderen & van der Meij, 2016, and references therein). These diminutive crabs (<1 cm) are known to reside in dwellings in the coral skeleton induced by the crabs (Potts, 1915; van der Meij & Hoeksema, 2013). Their feeding biology and as a result also the nature of their symbiotic relationship has been debated (Abelson et al., 1991; Kropp, 1986; Potts, 1915; Simon‐Blecher et al., 1999). A recent stable isotope study on three Atlantic cryptochirid species confirmed that these crabs mostly feed on coral tissue and/or mucus; hence, the obligate association to their scleractinian hosts involves a clear trophic relationship furthermore resulting in a strong host dependency (Bravo et al., 2022).

Cryptochirids occur worldwide, mostly on tropical shallow‐water reefs, but they have also been observed at depths below 500 m (Kropp & Manning, 1987). Currently, this family includes 53 species in 21 genera (WoRMS, 2023), yet several species complexes have recently been uncovered, and more species are awaiting description (Bähr et al., 2021; Xu et al., 2022). Gall crabs inhabit a broad range of corals from at least 66 genera (Castro, 2015). Their host specificity was used as a scheme to define cryptochirid genera in the past (Fize & Serène, 1957), but this has been overhauled by major taxonomic revisions of both corals and crabs (Fukami et al., 2008; Huang et al., 2014; Kropp, 1990; Terraneo et al., 2017). With the exception of the generalist species *Troglocarcinus corallicola* Verrill, 1908, all cryptochirid species are host specific; some at host species level, whereas other crab species associate with one or more closely related coral genera (e.g., Bähr et al., 2021; van der Meij, 2015; van der Meij et al., 2015; Xu et al., 2022).

It is noteworthy that the coral genera *Acropora* Oken, 1815 and *Porites* Link, 1807 are not among the more than 66 recorded coral host genera of gall crabs even though researchers (including the authors) have extensively examined them for the presence of cryptochirids (Fize & Serène, 1957; Kropp, 1988). *Acropora* and *Porites* are among the most abundant and diverse zooxanthellate coral genera on tropical reefs worldwide (Veron, 2000; Wallace, 1999) and arguably those with the most complex evolutionary and taxonomic history (Cowman et al., 2020; Terraneo et al., 2021). They also belong to the four coral genera with the highest symbiont fauna diversity (Stella et al., 2011: figure 5). Several brachyuran crab species inhabit *Acropora* corals (e.g., *Tetralia* spp. Dana, 1851), *Domecia acanthophora* (Desbonne in Desbonne & Schramm, 1867) and, to a lesser extent, also some predominantly branching *Porites* colonies (e.g., *Mithraculus sculptus* (Lamarck, 1818)) (Coen, 1988; Marin & Spridonov, 2007; van der Meij et al., 2022). There are few published records of brachyuran crabs in massive *Porites*. Coles (1982) reported on the domeciid crab *Cherusius triunguiculatus* (Borradaile, 1902) inhabiting small chambers in *Porites lobata* Dana, 1846, but most records in massive *Porites* constitute (non‐brachyuran) hermit crabs in the family Paguridae Latreille, 1802 (McLaughlin & Lemaitre, 1993).

Here, we report the unexpected finding of Cryptochiridae dwellings in *Porites rus* (Forskål, 1775) and *Porites* sp. in the central Red Sea. Gall crab specimens were identified based on morphology and DNA barcoding. The novelty of this crab–coral association is discussed in light of host specificity and interspecific coral aggression.

## 2 MATERIALS AND METHODS

During a dive on May 15, 2022, at Rose Reef (N 22.310498, E 38.886728) near Thuwal in the central Saudi Arabian Red Sea, scleractinian corals were examined for the presence of gall crab dwellings. Dwellings were observed in two different *Porites* colonies and photographed in situ. Subsequently, small pieces of the coral skeleton—including crab and dwelling—were collected for further processing in the laboratory at King Abdullah University of Science and Technology (KAUST). The crabs were extracted from their hosts, photographed under a stereo microscope (Leica 205A), and subsequently preserved in 75% ethanol. The remaining coral skeleton was bleached using sodium hypochlorite (<6%), rinsed with fresh water, dried, and subsequently photographed under the stereo microscope.

The egg mass of the female crab and the fifth pereiopod of the male were removed for DNA extraction and molecular analysis based on the cytochrome oxidase subunit 1 (COI, partial) barcoding gene (Folmer et al., 1994). DNA was extracted using the DNeasy Blood & Tissue Kit (Qiagen), and subsequently, polymerase chain reaction (PCR) was carried out with Qiagen Multiplex PCR Kit (1000) under the following conditions: 2.3 μL H_2_O (Omega Bio‐tek, Inc. Nuclease free Water), 2 μL of primers LCO1490 and HCO2198 (Folmer et al., 1994) respectively, 7.5 μL Qiagen Master Mix and 1.2 μL DNA template. The thermocycling protocol included the following steps: 95°C for 15 min, followed by 39 cycles of 95°C for 30 s, 47°C for 1 min and 72°C for 1 min and finalized by 10 min at 72°C (Eppendorf Mastercycler Pro S vapo.protect 6325). The PCR product was purified according to the protocol of Illustra Exostar OneStep Kit and sent for Sanger Sequencing at KAUST Bioscience Core Lab. Sequences are available on GenBank under accession number OQ430764 and OQ430765.

## 3 RESULTS

**FIGURE 1:**
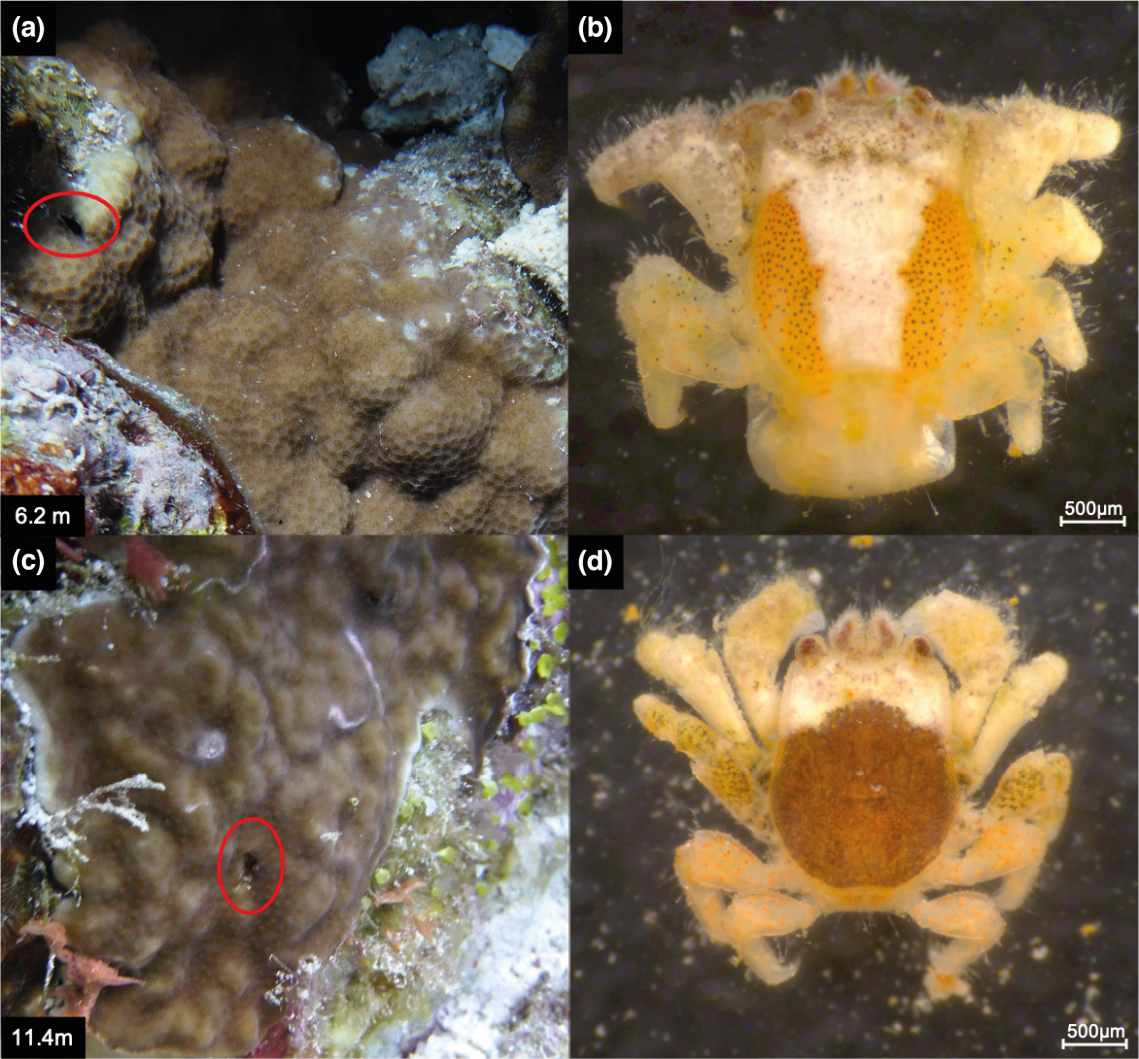
Two *Porites* corals colonies with crab dwellings at Rose Reef, off Thuwal (Saudi Arabia) and the cryptochirid specimens sampled from the respective colonies. (a) Encrusting *Porites* sp. colony with crescent‐shaped dwelling circled in red at a depth of 6.2 m. (b) Ovigerous female *Opecarcinus* SET.11 sampled from *Porites* sp. (a). (c) *Porites rus* colony encountered at 11.4 m depth; dwelling circled in red. (d) Male *Opecarcinus* specimen sampled from *Po. rus* (c). (b, d) Scaled to 500 μm.

**FIGURE 2:**
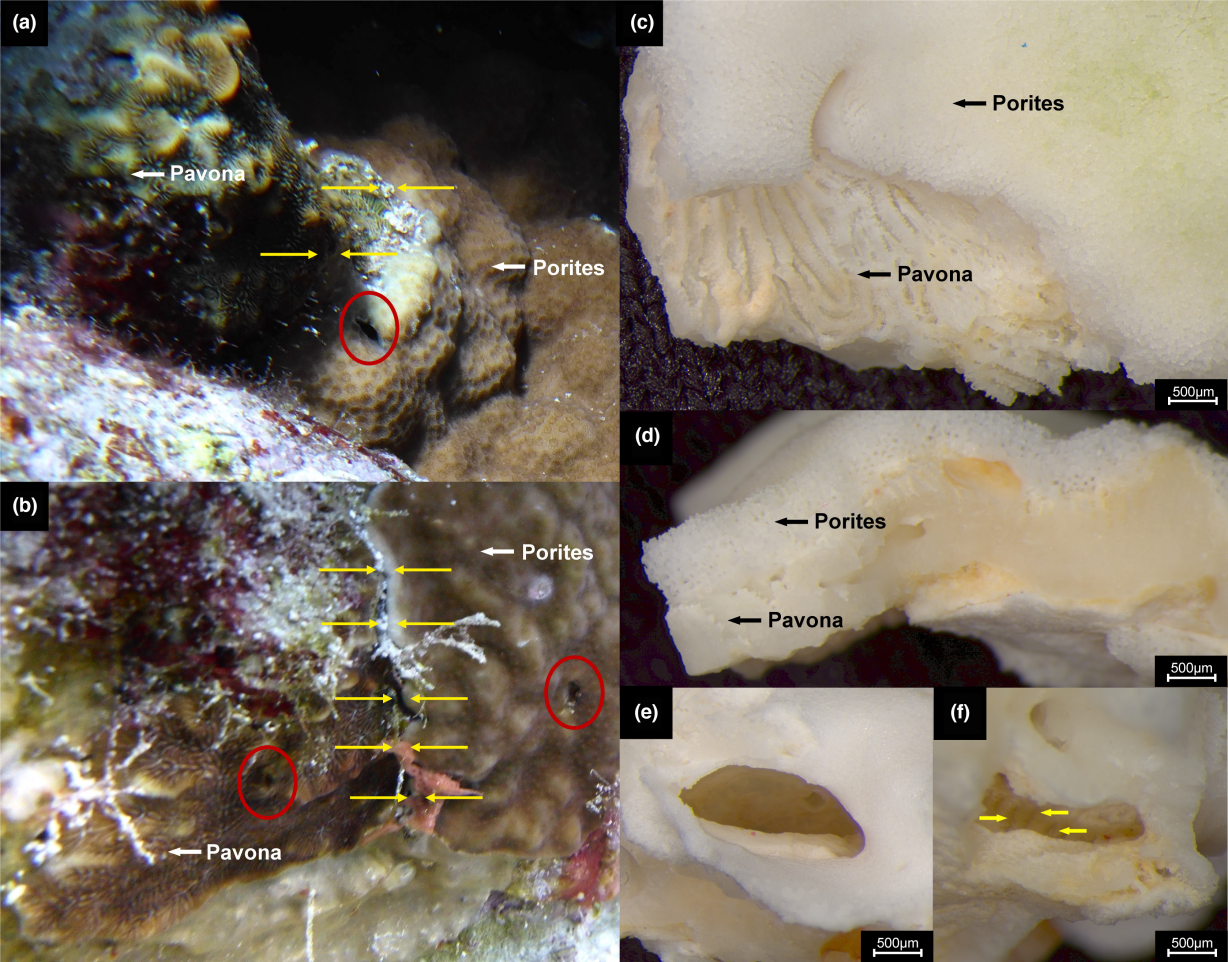
The two *Porites* colonies at Rose Reef, with adjacent *Pavona* colonies. (a) *Porites* sp. bordering (yellow arrows) *Pa. varians*. The gall crab dwelling is entirely surrounded by *Porites* tissue/skeleton. (b) *Porites rus* and adjacent *Pa. varians*. Yellow arrows indicate the bordering region with white to light‐pink colour where *Po. rus* is overgrowing *Pa. varians*. Additional crab dwelling on *Pa. varians* is circled in red. (c, d) Bleached *Po. rus* fragment with two different skeletal structures as indicated by the black arrows (c view from the top, d horizontal view). (e, f) Empty gall crab dwelling photographed in frontal (e) and anterior (f) orientation. Yellow arrows indicate the continuous radial elements of the *Pavona* skeleton.

Crescent‐shaped dwellings were observed in two different *Porites* colonies (Figure 1a,c). The corals were identified as *Porites* sp. (Figure 1a) and *Po. rus* (Figure 1c), based on the original species description and comparison with type material (Forskål, 1775) and scientific literature (Scheer & Pillai, 1983; Sheppard & Sheppard, 1991; Terraneo et al., 2021). The specimen identified as *Porites* sp. belongs to a species complex that contains, among others, *Po. lutea* Milne Edwards & Haime, 1851, *Po. lobata*, and *Po. solida* (Forskål), 1775 (Terraneo et al., 2021) and could not be identified down to species level based on morphology alone. The crab dwelling was located at the lateral colony margin of *Porites* sp. close to the bordering colony identified as *Pavona varians* (Verrill, 1864) (Figures 1a and 2a), and in case of *Po. rus*, it was observed in the center of the colony (Figures 1c and 2b). An ovigerous female gall crab was extracted from *Porites* sp. (Figure 1b), whereas a male was retrieved from *Po. rus* (Figure 1d). Both crabs have a vase‐shaped carapace that is longer than broad, widest posterior to midlength, deflected anteriorly, and convex in lateral view. The carapace has a transverse depression on the protogastric region, and the mesogastric region is mildly inflated. The female is bright orange with black speckles on the lateral sides of the dorsal carapace, whereas the cardio‐intestinal region is clear white. The cornea and the antennular peduncles (AP) dorsal side have a rust to dark rust shade (Figure 1b). In the male crab, the anterior third of the carapace is white, whilst the posterior two‐thirds of the carapace is dark rust. The eyes and AP are of similar hues as in the female crab. The merus of the third pereiopod is partially brown, whereas the fourth and fifth pereiopod have bright orange spots (Figure 1d). Based on their overall morphology and the crescent shape of their dwellings, the crabs were identified as belonging to the genus *Opecarcinus* Kropp and Manning, 1987 (Kropp, 1990; Kropp & Manning, 1987). The COI sequences of both collected specimens were blasted against the dataset of Xu et al. (2022) and had >99% identity with *Opecarcinus* SET.11, a species currently under description (T. Xu, G. Paulay, S. Vimercati, F. Benzoni, S. E. T. van der Meij, unpublished data).

Both *Porites* colonies were growing adjacent to *Pavona* Lamarck, 1801 colonies (Figure 2a,b). In the case of *Po. rus*, the peripheral belt of the contacting tissue showed light pink colouration (Figure 2b). Further examination in the laboratory of the bleached *Po. rus* fragments containing the crab dwelling suggested an overgrowth of the adjacent *Pavona* colony by *Po. rus*. Stereo microscope images revealed the presence of two different skeletal structures that are clearly separable based on gross morphologies (Figure 2c,d). The one on top, alive at the time of sampling, was characterized by the intermittent fusion of trabeculae and synapticulae, resulting in the typical porous skeletal structure of the genus *Porites*. Whereas the coral skeleton on the bottom, overgrown by the former, presented the typical thamnasteroid arrangement of the continuous radial elements of *Pavona* (Figure 2c,d). Furthermore, the photographs show that the opening of the dwelling is composed of porous skeletal structure (Figure 2e). In contrast, the inner and anterior parts are more strongly calcified and have visible continuous radial elements (Figure 2f), suggesting that *Po. rus* overgrew the dwelling present in the *Pa. varians* colony. In the same *Pa. varians* colony, another *Opecarcinus* dwelling is visible (Figure 2b).

## 4 DISCUSSION

To date, gall crabs have not been recorded as symbionts of *Porites* corals, making our observation of dwellings in *Po. rus* and *Porites* sp. the first of their kind. Earlier cryptochirid workers specifically mention the apparent absence of gall crabs in *Porites* (see e.g., Fize & Serène, 1957). The species‐rich genus *Porites* is, however, well‐known to harbor a diverse community of associated fauna, including mollusks, crustaceans, and polychaetes (Idris et al., 2022; Malay & Michonneau, 2014; Tsang et al., 2014; Zuschin et al., 2001). Decapod crabs are less commonly observed in association with encrusting or massive *Porites* corals as they seemingly prefer branching species for shelter (Marin & Spridonov, 2007; Patton, 1967; van der Meij et al., 2022). Hermit crabs of the genus *Paguritta* Melin, 1939 are the only decapods that inhabit dwellings in *Porites* that somewhat resemble cryptochirid dwellings in other encrusting or massive coral genera (McLaughlin & Lemaitre, 1993).

The gall crab dwellings observed in *Po. rus* and *Porites* sp. are morphologically identical to those of *Opecarcinus* in Agariciidae corals. Crabs of this genus typically reside in canopy‐like tunnels with a crescent‐shaped opening (Wei et al., 2013; van Tienderen & van der Meij, 2016: figure 4). While it is unknown if the coral or the crab is the main architect of the dwelling's morphology, observations of different cryptochirid species inhabiting the same coral genus suggest that the crabs make species‐specific contributions to the dwellings resulting in different dwelling morphologies. For example, crabs of the genera *Hapalocarcinus* Stimpson, 1859 and *Utinomiella* Kropp and Takeda, 1988 both inhabit *Pocillopora* Lamarck, 1816 corals; however, *Hapalocarcinus* crabs dwell in true, enclosed skeleton galls formed by the coral's branches, whereas *Utinomiella* crabs inhabit pits in the coral skeleton (Kropp, 1986). Interspecific adaptations for inhabiting different dwelling types have also been observed in other coral dwellers. Pagurid hermit crabs are known to either occupy empty tubes formed by *Spirobranchus* Blainville, 1818 worms, or to reside in self‐created boreholes in living corals (McLaughlin & Lemaitre, 1993).

The crabs collected from the *Porites* dwellings were identified as *Opecarcinus* SET.11 based on morphology and DNA barcoding. The circumtropical genus *Opecarcinus* has one Atlantic representative and another eight valid species occurring from the Red Sea to the Central Pacific Ocean, and their hosts extend to the eastern Pacific (Glynn & Ault, 2000; Kropp, 1989; van der Meij, 2014). Based on a global multimarker analysis, Xu et al. (2022) revealed high levels of undescribed species diversity in the genus. *Opecarcinus* SET.11 is among the most abundant *Opecarcinus* species in the Red Sea and has so far been recorded from *Pavona venosa* (Ehrenberg, 1834) and *Pa. varians* in the Red Sea (Xu et al., 2022; T. Xu, G. Paulay, S. Vimercati, F. Benzoni, S. E. T. van der Meij, unpublished data).

To date, there are no confirmed records of *Opecarcinus* in association with corals other than the Agariciidae Gray, 1847 (Kropp, 1989; van der Meij, 2014; Xu et al., 2022). After studying the in situ photographs in more detail, we noticed that there were *Pa. varians* corals adjacent to both of the *Porites* colonies (Figure 2a,b), one of the recorded hosts of *Opecarcinus* SET.11 in the Red Sea (Xu et al., 2022), in line with the observed host specificity in Cryptochiridae.

In both instances, the coral tissues of *Porites* and *Pavona* were directly touching (Figure 2a,b). The stereo microscope images of the bleached *Po. rus* fragments suggest that the adjacent *Pa. varians* colony was overgrown by *Po. rus* including the crab dwelling. Interspecific competition among scleractinian corals is a widespread phenomenon that shapes the ecology and bio‐constructional potential of coral reefs (Connell, 1973; Connell et al., 2004; Horwitz et al., 2017; Karlson, 1999; Sheppard, 1979). Lang and Chornesky (1990) identified eight different competitive mechanisms, including overgrowth. *Porites rus* seems to be particularly successful when competing with other *Porites* species (Idjadi & Karlson, 2007; Rinkevich & Sakai, 2001). Bleaching and the presence of pink color along the peripheral belt of the contacting tissue were specifically mentioned by Rinkevich and Sakai (2001), which is also visible in our *Po. rus* colony (Figure 2b). In both of the abovementioned studies, skeletal overgrowth was identified as the major driver of competition in their experiments. Such overgrowth is described as one of the main physical mechanisms adopted during inter‐ and intraspecific competition among scleractinian corals (Chadwick & Morrow, 2011). Based on these examples and our observations, we hypothesize that the *Pavona* colonies, including the gall crab dwellings, were likely overgrown by the adjacent *Porites* colonies, thus leading to a secondary association between *Opecarcinus* crabs and *Porites* corals.

How cryptochirids settle on their host coral is largely unknown, but their host specificity suggests fine‐tuned (chemical) cues are at play. Corals have been observed to aggressively fight off settlement of nonassociated fauna (Liu et al., 2016); hence, successful settlement of *Opecarcinus* larvae on *Porites* corals seems an unlikely scenario. When considering the overgrowth of *Pavona* by *Porites* as likely, it is important to acknowledge that there could be a time constraint due to the life span of gall crabs and the growth rate of corals. Little is known about the maximum age cryptochirids can reach. Kotb and Hartnoll (2002) speculate that the minimum life span of female *Hapalocarcinus marsupialis* s.l. (a species complex; see Bähr et al., 2021) crabs is in the range of 18–20 months based on their observations on gall stage durations in their pocilloporid hosts. Interestingly, a *H. marsupialis* s.l. female was sampled at Al Fahal reef (Red Sea) from a gall located at most basal part of a *Pocillopora* colony with an estimated age of at least 3 years based on its size (Erika Santoro, personal communications). The basal position of the gall suggests that the female is of similar age as its coral host. The pair‐forming coral‐dwelling hermit crab *Paguritta harmsi* (Gordon, 1935) inhabits self‐formed pits with a length of up to 98 mm in the *Astreopora myriophthalma* (Lamarck, 1816) on the Great Barrier Reef. Considering the absence of empty dwellings on *A. myriophthalma* colonies, two alternative hypotheses were postulated by the authors: (1) Pits are formed by juvenile crabs themselves, and (2) Vacant dwellings are occupied by new *Paguritta* individuals. The first hypothesis implies that the age of the hermit crab correlates with the growth rate of their colony since settlement of the hermit crab larvae. Based on the growth rates of *A. myriophthalma*, a 98‐mm‐deep dwelling translates to a life span of 7–13 years for *Pag. harmsi*. These findings indicate that coral‐dwelling decapods could be quite long lived and thus support our findings. The second hypothesis relies on an abundant supply of potential recruits; however, the close correlation between hermit crab length and pit depth makes a good fit between a new recruit and available dwellings unlikely (Patton & Robertson, 1980). Edmondson (1933) included a section of the host of *Cryptochirus coralliodytes* Heller, 1860 [as *C. rugusos*], showing a 6‐cm‐deep pit in which, the crab resided, affirming the findings of Patton and Robertson (1980) that coral‐dwelling decapods can be quite long‐lived.

In order to judge whether the overgrowth of the *Pavona* colonies by the two *Porites* colonies within the life span of the gall crabs is a likely scenario, the growth rates of *Porites* corals should be taken into account. A study on *Po. lutea* in Indonesia revealed average growth rates between 1.11 and 1.20 cm/year (Zamani & Arman, 2016). Based on the analysis of coral cores Klein and Loya (1991) found average linear growth rates of 7.48 and 5.68 mm/year for *Po. lobata* and *Po. columnaris* in the Gulf of Eliat, Red Sea. Yap and Molina (2003) estimated the growth rate of *Po. cylindrica* Dana, 1846, and *Po. rus* transplants based on the buoyant weight technique (Davies, 1989) over a time period of 2 years and reported on, respectively, a ninefold and sixfold increase. Lastly, in French Polynesia, *Po. rus* overgrew around 12% of the surface area of its inferior competitor *P. lobata* within a year (Idjadi & Karlson, 2007). Unfortunately, there are no data about the linear or radial extension rates for *Po. rus* in the Red Sea available, and thus the estimates made by these studies have to be interpreted with caution. In case of *Porites* sp., we can confidently assume that the neighboring *Pavona* was overgrown within the lifetime of the gall crab since the dwelling is in close proximity (<1 cm) to the border between the two colonies (Figure 2a). The crab dwelling on *Po. rus* is much further away from the bordering region between the colonies (Figure 2b). Based on the dwelling size, we estimate that it is approx. 4 cm away from the growth border of the colony. Nevertheless, given the observations about possible gall crab life span and the growth rates specified above, the distance of the dwelling to the colony edge seems plausible for an overgrowth scenario.

It is remarkable that the gall crabs were able to adapt to *Porites* as their secondary host, given the trophic relationship between gall crabs and corals (Bravo et al., 2022), and because the carbohydrate composition of mucus secreted by corals differs between species (Wild et al., 2010). In addition, the surface mucus layer of corals is known to harbor a diverse and species specific bacterial community that is sensitive to changes in environmental variables (Brown & Bythell, 2005; Osman et al., 2020). While there is some uncertainty about the crabs diet, they rely at least in part on coral tissue/mucus as a source of nutrition (Bravo et al., 2022; Kropp, 1986; Simon‐Blecher et al., 1999) and could thus be impacted by changes in mucus composition and associated microbial community. The *Porites*‐inhabiting *Opecarcinus* crabs seem to have successfully adapted to the altered conditions and are either capable of feeding on *Porites* tissue/mucus or can switch to alternative feeding modes. Moreover, the female crab collected from *Porites* sp. was egg‐bearing, hence still capable of obtaining the nutrients needed to invest energy in reproduction (Bähr et al., 2021).

Here, we reported on the first observation of *Opecarcinus* gall crab dwellings in *Porites* corals. Poritidae have never been reported as hosts for cryptochirid crabs, despite extensive search efforts by the authors and earlier scientists studying Cryptochiridae. The coral skeleton images revealed that *Porites* likely outcompeted their neighboring *Pavona* corals in the competition for space and unintendedly became “host” to the *Pavona*‐associated *Opecarcinus* crabs. Our findings suggest that *Opecarcinus* crabs are able to survive a substantial change in their obligate symbiotic relationship caused by interspecific coral aggression, and even thrive in their new surroundings with the female found capable of reproduction. Considering the trophic relationship between cryptochirids and their hosts, this feeding plasticity gives insights into the adaptive potential of gall crabs and hints at other processes (e.g., larval settlement) as drivers of host specificity.

## AUTHOR CONTRIBUTIONS

**Susanne Bähr:** Conceptualization (equal); data curation (lead); investigation (lead); visualization (lead); writing – original draft (lead); writing – review and editing (equal). **Sancia E. T. van der Meij:** Conceptualization (equal); investigation (equal); supervision (equal); validation (equal); writing – original draft (supporting); writing – review and editing (lead). **Tullia I. Terraneo:** Validation (supporting); writing – review and editing (equal). **Tao Xu:** Validation (supporting); writing – review and editing (equal). **Francesca Benzoni:** Funding acquisition (lead); resources (lead); supervision (equal); validation (equal); writing – review and editing (equal).

## FUNDING INFORMATION

This project was supported by funding from KAUST baseline research funds of F. Benzoni.

## CONFLICT OF INTEREST STATEMENT

The authors declare no conflict of interest.

## Data Availability

Sequences are deposited to GenBank under accession numbers OQ430764 and OQ430765.
